# Efficiency and safety of phenylephrine and tropicamide used in premature retinopathy: a prospective observational study

**DOI:** 10.1186/s12887-019-1757-3

**Published:** 2019-11-06

**Authors:** Atilla Alpay, Sılay Canturk Ugurbas, Cumhur Aydemir

**Affiliations:** 1 Department of Ophthalmology, Zonguldak Bülent Ecevit University, the School of Medicine, 67600 Zonguldak, Kozlu Turkey; 2 Department of Pediatrics, Division of Neonatology, Zonguldak Bülent Ecevit University, the School of Medicine, Zonguldak, 67600 Kozlu Turkey

**Keywords:** Retinopathy of prematurity, Mydriatics, Screening, Adverse events

## Abstract

**Background:**

To determine effects and side effects of topical application of phenylephrine 2.5% and tropicamide 0.5% combination in preterm infants.

**Methods:**

In this prospective observational study, 60 infants undergoing retinopathy of prematurity (ROP) screening were prospectively observed. Pupillary diameter, blood pressure, heart rate, and oxygen saturation were monitored before and after up to 24 h during ROP screening examinations.

**Results:**

The mean pupillary diameter 1 h after the instillation of drops was 5.58 ± 0.75 mm for both eyes. The mean systolic and diastolic pressure and oxygen saturation of infants did not change statistically until the end of the study. The average heart rate decreased by a mean of 4.96 beats/minute from the baseline following eye drops instillation. General condition deterioration, fall in oxygen saturation and bradycardia were observed in 4 infants that already had respiratory distress syndrome.

**Conclusion:**

The phenylephrine 2.5% plus tropicamide 0.5% drop is effective and safe as mydriatic combination for retinopathy of prematurity screening. In infants with an additional systemic disease such as respiratory distress syndrome, the side effects of mydriatic drops may be more common. Such babies should be kept under close observation.

**Trial registration:**

The trial was retrospectively registered on 28 February 2018. The ClinicalTrials.gov Identifier is NCT03448640.

## Background

Today, advances in perinatal and neonatal care have increased the survival of extremely preterm infants and the number of infants undergoing retinopathy of prematurity examinations [1, 2]. Mydriasis should be sufficient for a complete fundus examination. Acceptable mydriasis can be formed with all the current available drugs provided that they are used in an adequate concentration and dosage. Anti-parasympathetic (tropicamide, and cyclopentolate) and sympathetic agonist (phenylephrine), either alone or in various concentrations and combinations, are commonly used to mydriasis. The goal of these combinations is to perform ideal dilatation to allow peripheral retinal examination with minimal side effect. Many previous studies have investigated the effects of different combinations of different doses of mydriatics to achieve this goal and did not report exactly identical results of the same mydriatic combinations [3–8]. Nevertheless: a combination of phenylephrine 2.5% plus tropicamide 0.5% ophthalmic drops has generally been reported as safe. But, neonatologists reported that some infants had suffered from vomiting, bradycardia, hypotonia, and aspiration risk during the 30 to 60 min following the fundus examination conducted in neonatal intensive care units. The mean effects of mydriatic drops on all infants have been reported in previous studies. However, infants whose general condition had already been disturbed and undergoing R examinations were not addressed separately. Our aim in this study, after ROP examination, is to determine the characteristics of babies whose general condition deteriorated. Therefore, we investigated the pupil dilation effects and side effects of the triple instillation of phenylephrine 2.5% plus tropicamide 0.5% ophthalmic drop combination, which we routinely use in the retinopathy of the prematurity (ROP) examination.

## Methods

This prospective observational study was approved by the Bulent Ecevit University Clinical Research Ethics Committee. All procedures adhered to the tenets of the Declaration of Helsinki. Informed written consent was obtained from all the participants’ parents.

Sixty premature infants were included in this study and were undergoing their first eye examination for ROP. Infants with any of the following were excluded from the study: any developmental ocular and/or systemic anomalies (such as cardiovascular, renal, and gastrointestinal), drug use that can affect vital values, unstable general condition, food intolerance and recurrent vomiting.

The infants’ oral intake was halted just before the instillation of the drops and up to 1 h after the fundus examination. The gestational age of the infants, and the postmenstrual age at the time of examination were recorded. Vital factors such as heart rate (HR), blood pressure (BP), and oxygen saturation were monitored and recorded by using the Infinity® Vista XL Patient Monitor.

One drop of the combination of phenylephrine 2.5% and tropicamide 0.5% was instilled 3 times, every 10 min apart and the cutaneous absorption was minimized by wiping away the excess fluid from the periocular area [9]. The fundus examination was performed 60 min after the first instillation. Fundus examination was performed as follows. One drop of 0.5% proparacaine hydrochloride was instilled to each eye, before the speculum was placed. The horizontal pupillary diameter measurement was performed just before the first instillation of drops and then before fundus examination with a small ruler. The examination was then performed using a pediatric scleral depressor and indirect ophthalmoscopy with a 20 diopter lens.

Vital factors were examined 7 times during the study. The first 3 measurements were made just before the instillation of drops and recorded. Follow up measurements were taken 10 min after the third measurement, just before the fundus examination, 30 min and 60 min after the fundus examination and recorded.

The nursing staff was also asked to record any episodes of vomiting or intolerance to feedings and if there was any deterioration in the vital sings within 24 h after the fundus examination, except for the specified periods.

The statistical analyses of the study were performed using SPSS 19.0 package program. The descriptive statistics of continuous variables were shown via median, minimum and maximum values; categorical variables were shown via frequency and percentage. Shapiro Wilk test was used for testing normality. The values obtained before and after the instillation of drops were compared by paired *t-*test and Wilcoxon signed-rank test. The results were evaluated at 95% confidence interval and *p* < 0.05 was considered significant.

## Results

Sixty premature infants were involved in this study. The birth weight, mean gestational age at the birth and the postmenstrual age at the time of examination were 1451 ± 37 g, 29 ± 2.2 weeks, and 32.86 ± 1.2 weeks, respectively. The mean pupillary diameter was 1.82 ± 0.44 mm before the instillation of drops and 5.58 ± 0.75 mm just before the examination. The examination lasted 4–7 min. There was no complication such as bradycardia, apnea or vomiting during the examination. Eight infants were requiring oxygen at the time of examination. In the follow-up visits, none of these babies had any deterioration in vital signs.

**Fig. 1:**
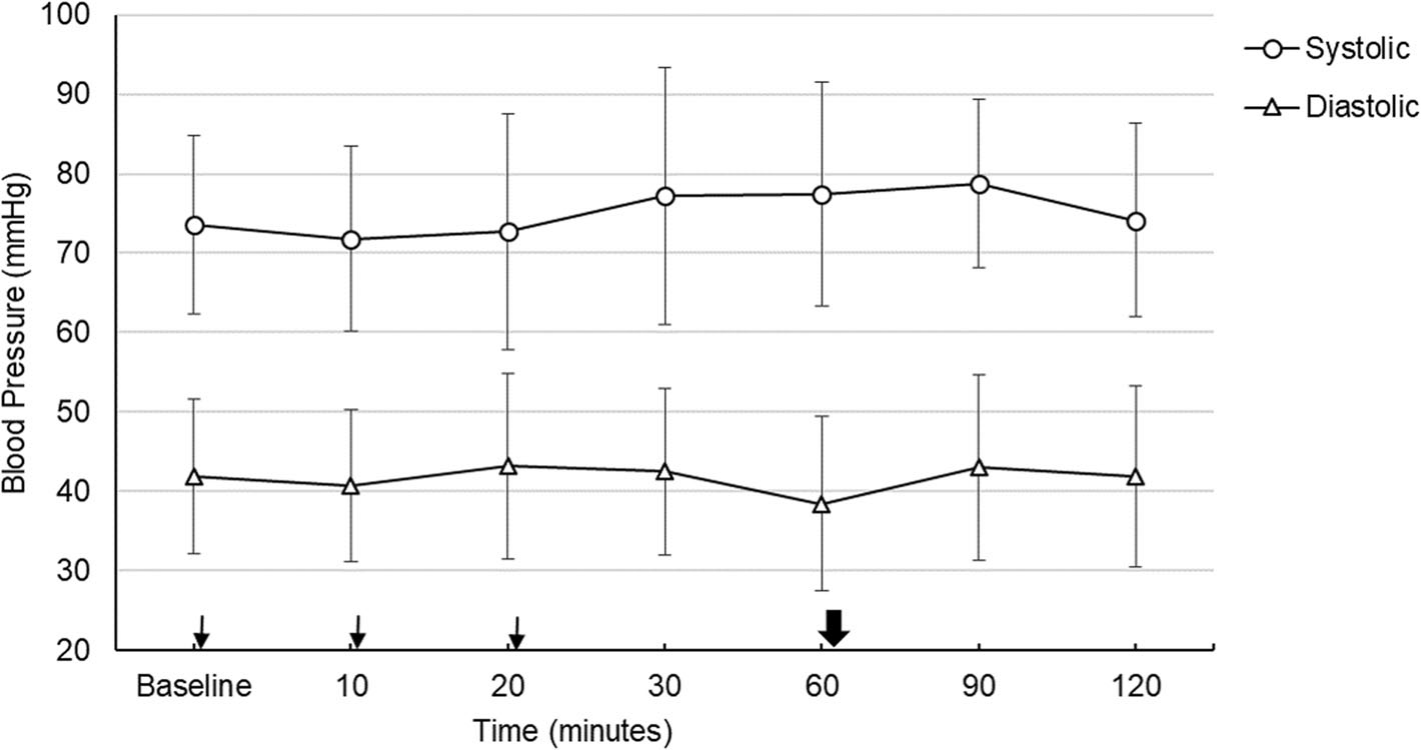
Mean systolic and diastolic blood pressure changes during the study. Thin arrows; shows the time of instillation of eye drops immediately after taking vital values. Bold arrow; shows the time of fundus examination immediately after taking vital values

The mean systolic pressure and diastolic pressure of the infants was 73.5 ± 11.2 mmHg and 41.8 ± 9.7 mmHg, respectively immediately before the administration of the mydriatic drops. After the instillation of mydriatic drops, there was no statistically significant change in the averaged mean values of both the systolic and diastolic pressure levels until the completion of the study (*p* = 0.758, *p* = 0.989 respectively). The changes in mean BP are shown in Fig. 1.

**Fig. 2:**
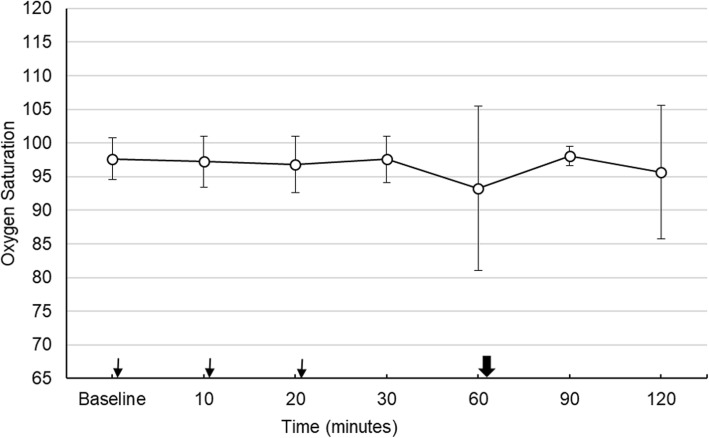
Mean pulse rate during the study. Thin arrows; shows the time of instillation of eye drops immediately after taking vital values. Bold arrow; shows the time of fundus examination immediately after taking vital values

The average HR of the infants was 143.3 ± 12.7 beats/min. Immediately before the administration of the eye drops. The average HR decreased by 4.96 beats/min below the basal value 10 min after the first eye drops instillation. This decline was statistically significant (*p* = 0.02). The highest decrease (6.64 beats/min) in average HR was seen in the 4th measurement just before fundus examination. Excluding the baseline records, the range of deviation among the subsequent readings was within 3.6 beats/minute and there was no significant difference between these values. Thirty minutes after the fundus examination, the average HR was slightly increased (1.78 beats/min) and in the last measurement decreased again (3.28 beats/min). Figure 2 shows the changes in mean HR recorded during the examination.

**Fig. 3:**
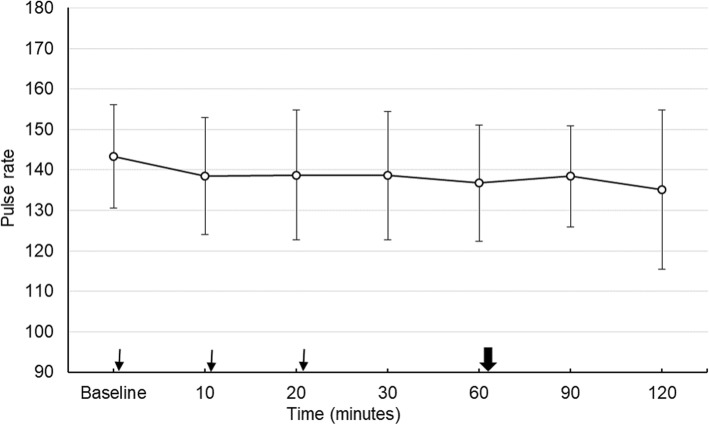
Median oxygen saturation during the study. Thin arrows; shows the time of instillation of eye drops immediately after taking vital values. Bold arrow; shows the time of fundus examination immediately after taking vital values

There was a slight decrease in the level of average oxygen saturation after the installation of the mydriatic drops. During the study period there were small change in the average oxygen saturation level. İt was constant between 93 and 98%. The changes were not statistically significant. The lowest average value of oxygen saturation was 93.3 ± 12%. This value was obtained in the fourth measurement just before the fundus examination and was not statistically significant compared to the basal measurement (*p* = 0.08). Thirty minutes after the fundus examination, it increased again to 98 ± 1.4% and in the last measurement, it fell back to 94.7 ± 15%. Figure 3 shows the oxygen saturation changes during the whole course of the study.

Four infants who were previously diagnosed with respiratory distress syndrome (RDS) showed general condition deterioration 40–70 min after fundus examination. This deterioration was seen especially in the HR and oxygen saturation. The mean HR, oxygen saturation, systolic pressure and diastolic pressure of these infants were 91.7 ± 20.1 beats/min, 55.5 ± 29.2%, 68.7 ± 18.5 mmHg and 45.2 ± 14.6 mmHg, respectively. Food intolerance and vomiting were not observed within 24 h after the instillation of eye drops.

## Discussion

To achieve a satisfactory fundus examination, the pupil diameter should be 5 mm or larger. If the pupil dilatation is weak, the duration of the examination is prolonged and the infants become more stressed. Phenylephrine and tropicamide or cyclopentolate have an additive effect and the combined use of these drugs is twice as effective as their use alone [10–12]. Using only one drop of phenylephrine 2.5% and one drop of tropicamide 0.5% has been shown to be safe and effective in eyes with light irises [13, 14]. But dilatation of highly pigmented (darker) irises more difficult than lightly pigmented irises, because the pigment binds the drug molecules and may require higher drug concentration or more frequent administration of mydriatic agents [3]. The majority of the infants in our study group had darkly pigmented irises. Our mydriatic regimen of the triple instillation of phenylephrine 2.5% and tropicamide 0.5%, has been shown to be effective and, provided consistent satisfactory mydriasis as in Khoo et al’ [10] study.

Phenylephrine is a selective α1-adrenergic receptor agonist that affects the sympathetic system. It also affects β-receptors at very high doses. When phenylephrine binds β-receptors, a rise in BP and reflex bradycardia is observed [15, 16]. Tropicamide and cyclopentolate are parasympatholytic drugs. They work by blocking the cholinergic receptors that exhibit a small effect on the cardiopulmonary system. They have a similar side effect profile, which includes oxygen desaturation, apnea, feeding intolerance, delayed gastric emptying, transient paralytic ileus, tachycardia, seizures, and psychosis [9, 17–20].

Drug side-effects are more common in premature infants because of they have very small body weight, small blood volume and immature drug metabolism [21]. After application of mydriatic eye drops absorb into the systemic circulation from the cornea, conjunctiva and nasal mucosa, and may alter the BP, HR, and oxygen saturation [3, 4, 22, 23].

Many studies have reported an increase in BP when using phenylephrine 2.5% combined with tropicamide or cyclopentolate 0.5% or 1% [3–5, 7, 8, 24]. On the other hand, Bolt et al. [13] did not observe a significant increase in BP after the instillation of the phenylephrine 2.5% plus tropicamide 0.5% combination, and they recommended this combination in order to achieve a sufficient diagnostic mydriasis without any systemic side effects in preterm infants. In our study, there was no statistically significant change in the BP. As in our study, many others [8, 14, 25] did not detect a rise in BP using cyclopentolate 0.5% alone, cyclopentolate 0.5% + tropicamide 0.5%, cyclopentolate 0.5% + phenylephrine 2.5%, Cyclopentolate 0.2% + Phenylephrine 1%, tropicamide 1% + phenylephrine 2.5% eye drops. These differences in studies may be related to the general condition of premature infants in the study groups or the care standards in the neonatal intensive care unit.

The HR increase significantly has been observed in many studies using different combinations and concentration of phenylephrine, tropicamide and cyclopentolate [4, 5, 26]. Bolt et al. [13] reported that cyclopentolate causes a significant increase in HR, whereas 2.5% phenylephrine and 0.5% tropicamide do not lead to a significant change. But, other some studies [8, 24] did not find significant changes in HR after the phenylephrine 2.5% and cyclopentolate 0.5% eye drop instillation. In our study, ten minutes after the first installation of the mydriatics, the HR decreased by an average of 4.96 beats/minute. This decrease was statistically significant. The combination of mydriatic drops seems to affect the HR most frequently than other vital values, but this effect was clinically insignificant. At the examination following the 3rd drop instillation, the average HR dropped a little more. Thirty minutes after the examination, pulse rate increased again. The reason for this increase is probably the stress caused by the examination. The fall in HR has been reported in a limited number of previous studies [3, 10].

The effects of mydriatics on oxygen saturation have been reported in many studies. The results of literature is conflicting, as some studies have concluded a decline in oxygen saturation, whereas others have found no harmful effects [23, 24]. Mitchell et al. [19] observed a decrease in oxygen saturation in 78% of the infants subsequently the triple instillation of the combination of cyclopentolate hydrochloride 0.2% and phenylephrine hydochloride 1% (Cyclomydril®, Alcon, Ft. Worth, TX) eye drop. Neffendorf et al. [25] studied the effect of 3 consecutive instillation of phenylephrine 2.5% and cyclopentolate 0.5% in 623 eye examinations in the 24-h period after the ROP screening and reported that this combination was efficacious and safe except for in 5 infants with respiratory problems such as bronchopulmonary dysplasia and respiratory distress syndrome of the newborn. Resembling to this observation, Mitchell et al. [27] reported bradycardia and hypoxia 1 h after cyclomydril eye drops instillation in preterm infants whose were sicker and on some type of respiratory support. They investigated the blood level of cyclomydril eye drops 1 h after the instillation. Cyclopentolate was detected in 15 of 18 infants, while phenylephrine was not measurable in any patients. They attributed the side effects to the cyclopentolate blood levels in risky patients. These studies show that a physiologically susceptible group of infants with respiratory diseases is at risk for bradycardia and oxygen desaturation due to inadequate metabolization of cyclopentolate. In our study too, average oxygen saturation level decreased after the instillation of mydriatic drops but did not show any significant change. However, hypotonia, bradycardia and significant decrease in oxygen saturation were observed in four infants, approximately 1 h after the fundus examination. These infants already had respiratory problems such as bronchopulmonary dysplasia and respiratory distress syndrome of the newborn. This deterioration could also be attributed to stress of scleral depression rather than mydriatic eye-drop combination. Other adverse effects such as abdominal distention, cyanosis, and clinical signs of seizure activity were not observed in our study.

Fundus examination is a very stressful and painful procedure for infants [28]. It may be considered that some infants whose general condition is impaired are more devastated than the ROP examination. Many studies have reported that premature infants who are impaired general condition may develop complications such as intraventricular hemorrhage and apnea when they are confronted with a stressful situation [27, 29–31]. Placing eye speculation and applying pressure to the eyeball with a depressor is a very uncomfortable process. This procedure may lead to traction on the rectus muscle, stimulation of vagal neurons and bradycardia [19]. We did not examine the effects of fundus examination directly, however thirty minutes after the fundus examination, an increase in HR and oxygen saturation was observed followed by a decrease in subsequent measurements. This observation is probably due to the stress induced during the fundus examination.

Both anticholinergic agents’ tropicamide and cyclopentolate have a similar side effect profile. Three times instillation of phenylephrine 2.5% and cyclopentolate 0.5% is often used for ROP screening. This procedure is advised by the Royal College of Ophthalmologists [32]. But the average onset time for tropicamide is shorter than that of cyclopentolate, and the trecovery time for tropicamide is almost five times faster than that of cyclopentolate [17, 33, 34]. For all these reasons, use of tropicamide instead of cyclopentolate may be safer especially in weak and sickly infants.

## Conclusion

In our study, during the 24-h period following the ROP examination, 93.3% (56/60) of infants did not have a significant systemic or ocular complication. Three times repetition of instillation of phenylephrine 2.5% and tropicamide 0.5% combination can be considered effective and safe for ROP examinations of premature infants hospitalized in the intensive care unit. Infants with additional diseases, such as RDS, should be monitored more carefully after the ROP examination. Efforts should be made to ensure adequate mydriasis to prevent stress resulting from time consuming examinations. The use of tropicamide in place of cyclopentolate may be considered in mydriatic regimens because the duration of action of tropicamide begins earlier and ends sooner than cyclopentolate. Nurses should be instructed to wipe off the excessive drops which spill out of the eye after instilling as the absorption from the eyelid skin can lead to periocular blanching and systemic complications [35].

The fatigue and the accumulative effects of stress and pain may contribute to adverse effects particularly in infants who have additional illnesses such as respiratory distress syndrome. Anticholinergics such as tropicamide or cyclopentolate may also contribute to the emergence of side effects. Research is needed on factors such as medical history, general condition, fatigue and the cumulative effects of stress and pain and to compare the side effects of mydriatic regimens containing tropicamide versus cyclopentolate. Further examinations with large samples are needed to fully understand the adverse effects of the ROP examination in infants with additional disabilities. In such infants, there is a need for new studies to determine the dosage of mydriatic drops, combinations and their frequency of installation.

## Data Availability

The datasets used during the current study available from the corresponding author on reasonable request.

## Abbreviations

BP: Blood Pressure
HR: Heart rate
RDS: Respiratory distress syndrome
ROP: Retinopathy of prematurity
